# Diabetic Ketoacidosis Fluid Therapy Algorithm in the Golden Hours: Iatrogenic Hyperchloremic Acidosis Instead of Unmeasured Anion Acidosis

**DOI:** 10.3390/jcm14124125

**Published:** 2025-06-11

**Authors:** Zeynep Tugce Sarikaya, Bulent Gucyetmez, Duran Ozdemir, Behiye Dogruel, Aykut Ayyildiz, Jozef Kesecioglu, Lutfi Telci

**Affiliations:** 1 Department of Anesthesiology and Reanimation, School of Medicine, Acıbadem Mehmet Ali Aydınlar University, 34752 Istanbul, Turkey; tugcesrky@gmail.com (Z.T.S.); bulentgucyetmez@gmail.com (B.G.); 2 General Intensive Care, Acıbadem Bakırkoy Hospital, 34140 Istanbul, Turkey; drdrn14@gmail.com; 3 General Intensive Care, Acıbadem Atakent Hospital, 34303 Istanbul, Turkey; behiye.dogruel@acibadem.com; 4 General Intensive Care, Acıbadem Maslak Hospital, 34457 Istanbul, Turkey; aykut.ayyildiz@acibadem.com.tr; 5 Department of Intensive Care Medicine, University Medical Center, 3584 CX Utrecht, The Netherlands; 6 General Intensive Care, Acıbadem International Hospital, 34149 Istanbul, Turkey; telci@istanbul.edu.tr

**Keywords:** diabetic ketoacidosis, hyperchloremia, base-excess chloride, fluid

## Abstract

**Background/Objectives**: In diabetic ketoacidosis (DKA), absolute insulin deficiency and elevation of counter-regulatory hormones may cause osmotic diuresis and water and electrolyte loss, which may lead to dehydration and renal failure. Fluids with high Na content are preferred in the DKA fluid therapy algorithm due to the association of Na with β-Hydroxybutyrate (β-HB) and the renal excretion of Na-β-HB. However, these fluids may cause hyperchloremic metabolic acidosis due to their high chloride concentration. In the literature, base-excess chloride (BE_Cl_) has been suggested as a better approach for assessing the effect of chloride on acid–base status. Our aim in this study was to investigate the effect of fluids with BE_Cl_ values less than zero versus those with values equal to or greater than zero on the metabolic acid–base status in the first 6 h of DKA. **Methods**: This retrospective study included DKA cases managed in the tertiary intensive care units of five hospitals in the last 10 years. Patients were divided into two groups according to the Na-Cl difference of the administered fluids during the first 6 h of treatment: Group I [GI, fluids with Na-Cl difference = 0, chloride-rich group] and Group II [GII, fluids with Na-Cl difference > 32 mmol, chloride non-rich group]. Demographic data, blood gas analysis results, types and amounts of administered fluids, urea–creatinine values, and urine ketone levels were recorded. **Results**: Thirty-five patients with DKA in the ICU were included in the study (GI; 22 patients, GII; 13 patients). There was no difference between the patients in the two groups in terms of age, gender, and LOS-ICU. According to the distribution of the administered fluids, the main fluid administered in GI was 0.9% NaCl, whereas in the GII, it was bicarbonate, Isolyte-S, and 0.9% NaCl. In GI, the chloride load administered was higher; the BE_Cl_ level of the fluids was lower than in GII. At the end of the first 6 h, although sodium and strong ion gap values were similar, patients in GI were more acidotic due to iatrogenic hyperchloremia and, as a result, were more hypocapnic than GII. **Conclusions**: In conclusion, administering chloride-rich fluids in DKA may help reduce unmeasured anion acidosis. Still, risks cause iatrogenic hyperchloremic acidosis, which can hinder the expected resolution of acidosis and increase respiratory workload. Therefore, it is suggested that DKA guidelines be revised to recommend an individualized approach that avoids chloride-rich fluids and includes monitoring of metabolic parameters like Cl and BE_Cl_.

## 1. Introduction

DKA is a life-threatening metabolic disorder that requires rapid intervention. Intensive fluid administration and blood sugar regulation within the first 24 h have a significant impact on survival. The fact that the most frequently debated issues are still the type of fluid and insulin infusion highlights the need for clarification and potential revision [1]. Although 0.9% NaCl remains the most commonly used fluid in DKA treatment, recent studies have raised concerns about its association with hyperchloremic metabolic acidosis and prolonged intensive care unit stays [2]. Alternatives to 0.9% NaCl are being researched that could positively contribute to the survival of DKA patients in intensive care. 

In the diabetic ketoacidosis (DKA) fluid therapy algorithm, recommended fluids and their amounts for the first 6 h, which we prefer to name “golden hours,” are defined in detail [3]. This fluid therapy aims to restore circulatory volume, clearance of ketones, and correction of electrolyte imbalance [4]. For these purposes, the only recommended fluid is still 0.9% NaCl, which contains 154 mmol/L Na and 154 mmol/L Cl. Pathophysiologically, DKA leads to unmeasured anion (UA) acidosis due to increased β -hydroxybutyrate (β-HB) [5]. β-HB is excessively produced in the liver during DKA and dissociates into β-HB anion (β-HB^−^) and H^+^ in the blood [6]. β-HB^−^ is laboured to excrete from kidneys by linking up Na, whereas CO_2_ is produced by linking up H^+^ with HCO_3_^−^ and is expelled via the lungs. Even though 154 mmol/L Na in 0.9% NaCl may ease β-HB^−^ excretion from the kidney, administering the same amount of Cl will cause hyperchloremia and hyperchloremic acidosis in the blood, another form of electrolyte imbalance. This study investigated the hyperchloremic effects of the recommended fluids during the golden hours of the DKA fluid therapy algorithm.

## 2. Materials and Methods

### 2.1. Ethics Approval

This retrospective study was approved by the Acıbadem Mehmet Ali Aydınlar University Local Ethics Committee, Istanbul, Turkey (Decision No: ATADEK-2020/02/27).

### 2.2. Definition of DKA

According to guidelines, DKA was defined as blood glucose > 200 mg/dL, HCO_3_ < 15 mmol/L, pH < 7.3, and urine ketones ≥ 2(+) [3].

### 2.3. Patients

**Figure 1:**
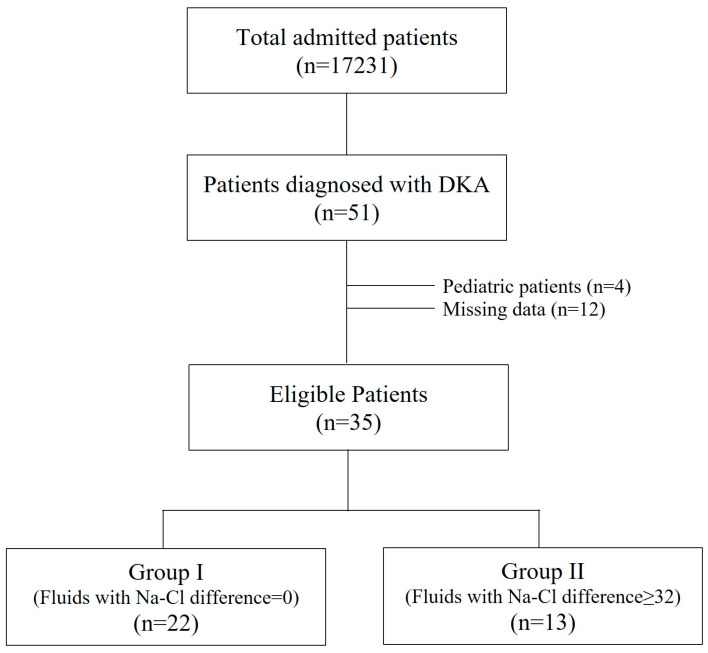
Study flowchart.

All admitted patients in the last 10 years at five tertiary intensive care units (ICU) were examined. Patients who were >18 years old with DKA were included in the study. Paediatric patients and patients with missing data were excluded (Figure 1). None of the patients had chronic renal failure. All were insulin-dependent DM patients. All adult DKA patients were divided into two groups: Group I (GI) was administered fluids with Na-Cl difference = 0, and Group II (GII) was administered fluids with Na-Cl difference > 32 mmol (Figure 1). Base-excess chloride (BE_Cl_) was used to detect hyperchloremia in patients [6].

### 2.4. Collected Data

For all patients, the following data were collected: age (years), sex, body mass index (BMI) (kg/m^2^), Charlson comorbidity index (CCI), APACHE II, SOFA score, arterial pH, PaCO_2_ (mmHg), HCO_3_ (mmol/L), standard base excess (SBE) (mmol/L), Na (mmol/L), K (mmol/L), Cl (mmol/L), BE_Cl_ (mmol/L), lactate (mmol/L), Strong Ion Gap (SIG) (mmol/L) at ICU admission and at the 6th hour; urea (mg/dL), creatinine (mg/dL), and urine ketones at ICU admission and at the 24th hour; and the length of ICU stay (LOS-ICU).

SIG and BE_Cl_ were calculated for each patient using the following formulas [6,7]:

SIG = (Na + K + Ca + Mg-Cl-lactate) − (0.003 × P_a_CO_2_ × 10^(pH-6.1)^) − (Alb(g L^−1^) × [0.123 × pH-0.631]) − (P_i_ (mmol L^−1^) × [0.309 × pH-0.469])

BE_Cl_ = Na-Cl-32 mmol

Normal values for both SIG and BE_Cl_ were accepted as zero [6,8]. SIG > 0 indicated UA acidosis. BE_Cl_ < 0 indicated hyperchloremia, whereas BE_Cl_ > 0 indicated hypochloremia [6].

Blood gas data were acquired using an ABL 800 (Radiometer, Denmark, Copenhagen) blood gas device, which employs ion-selective electrodes. Alb, Mg, P_i_, urea, and creatinine were acquired using a Cobas C 303 device (Roche, Rotkreuz, Switzerland).

### 2.5. Statistical Analysis

Descriptive data are presented as mean ± sd, median (quartiles), and percentages. The Shapiro–Wilk test was used to assess normality. Student’s *t*-test, Mann–Whitney U, and chi-square (Fisher’s exact) tests were used to compare groups. Pearson’s correlation test was used to measure the LOS-ICU correlation. A multivariate linear regression model was used to estimate the effects of 14 parameters on LOS-ICU. The estimated power of this study was detected as 0.99, calculated using an independent samples *t*-test for BE_Cl_ at the 6th hour between groups ( group sizes were 21 and 13, respectively, and the mean and standard deviation differences in BECl levels at the 6th hour were 9.0 mmol/L and 3.0 mmol/L, respectively, with α = 0.5). SPSS version 29 was used for all statistical analyses, and a *p*-value < 0.05 was considered statistically significant.

## 3. Results

### 3.1. At the ICU Admission

The incidence of DKA was 3.0 (51 of 17231) per 1000 patients, and 35 patients with DKA were included in the study (GI [%0.9 NaCl] = 22 and GII [fluids with Na-Cl difference > 32] = 13) (Figure 1).

Patient characteristics, as well as blood gas and laboratory parameters, were similar at ICU admission (Table 1).

All patients had a pH ≤ 7.25 at ICU admission. The number of patients with pH ≤ 7.0 at ICU admission was 6 (27.3%) in GI and 6 (46.2%) in GII (*p* = 0.262). The number of hyperchloremic (BE_Cl_ < 0) patients at ICU admission was 15 (68.2%) in GI and 12 (92.3%) in GII (*p* = 0.114). Since there were no differences in clinical or biochemical parameters between the groups, there were no confounding factors that could affect fluid selection or outcomes.

### 3.2. Administered Fluids in the First 6 h (‘Golden Hours’)

In GI, 0.9% NaCl was administered to 21 (95.5%) patients. Other fluids used included 5% dextrose and 0.9% NaCl, 0.45% NaCl, 5% dextrose and 0.45% NaCl, 5% dextrose, 6% HES, and albumin (Table 2.) The Na-Cl difference for all these fluids was zero. On the other hand, in GII, Isolyte and Isolyte-S, which are defined as balanced fluids, were administered to four (30.8%) patients, whereas 935 mL 0.45% NaCl plus 65 mL NaHCO_3_ combination, which is a craftwork fluid, was administered to five (38.5%) patients (Table 2). The Na-Cl differences for these balanced and craftwork fluids were 37, 43, and 50 mmol, respectively (Table 2).

### 3.3. At the End of the Golden Hours

At the end of the first 6 h, the total amount of administered fluids, intravenous bolus NaHCO_3_, KCl, insulin, Na, and urine output were similar in both groups (*p* = 0.699, *p* = 0.389, *p* = 0.511, *p* = 0.933, *p* = 0.091, and *p* = 0.448, respectively) (Table 3). However, the total amount of administered Cl was significantly higher; the Na-Cl difference of administered fluids was significantly lower in GI than GII (*p* = 0.016 and *p* < 0.001, respectively) (Table 3). Moreover, pH, PaCO_2_, HCO_3_, SBE, and BE_Cl_ were significantly lower; Cl was significantly higher in GI than GII (*p* < 0.001, *p* = 0.010, *p* < 0.001, *p* = 0.002, *p* < 0.001, and *p* < 0.001, respectively) (Table 3). As for Na and SIG, there was no significant difference between the groups (*p* = 0.622 and *p* = 0.408) (Table 3).

**Figure 2:**
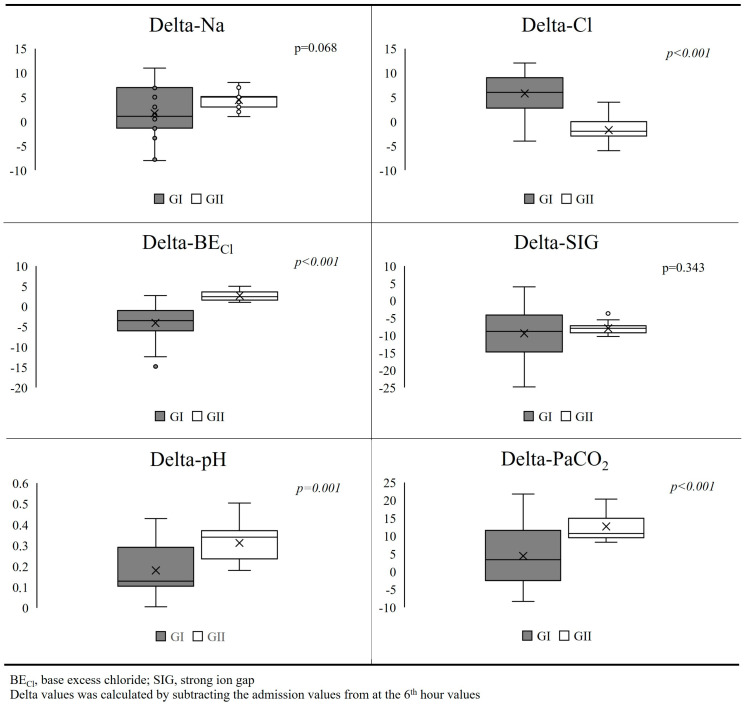
Comparison of delta values between groups.

Delta values of Na and SIG, which were calculated by subtracting the admission value from the 6th-hour value, were similar in both groups (*p* = 0.068 and *p* = 0.343) (Figure 2). However, delta-Cl was significantly higher, and delta-pH, delta-PaCO_2_, and delta-BE_Cl_ were significantly lower in GI than GII (*p* < 0.001, *p* = 0.001, *p* < 0.001, and *p* < 0.001, respectively) (Figure 2).

There were negative and positive correlations between delta-pH, LOS-ICU, and delta-Cl and LOS-ICU (r = −0.42 and r = 0.67; *p* = 0.014 and *p* < 0.001, respectively). No correlation was observed between other blood gas parameters and LOS-ICU. In the multivariate linear regression model, LOS-ICU increased only with increases in delta-Cl and delta-lactate levels (*p* = 0.031 and *p* = 0.038, respectively) (Table 4).

## 4. Discussion

This study shows that using fluids with zero Na-Cl difference, as recommended in the DKA guidelines, causes hyperchloremic acidosis even in the first 6 h, despite improving SIG levels during that period. On the other hand, fluids with ≥32 mmol Na-Cl difference led to better metabolic status and CO_2_ levels, which may also have improved hyperventilation in the same period, as they do not cause hyperchloremic acidosis.

Fluid therapy in DKA aims to restore circulatory volume, eliminate ketones, and correct electrolyte imbalances [3]. The recommended fluids in the guidelines should be discussed to determine whether they serve these three purposes.

### 4.1. Restoration of Circulatory Volume

Due to glucosuria, osmotic diuresis frequently leads to dehydration in all patients, and guidelines define how volume replacement is managed in detail. Furthermore, there is no debate in the literature about the recommended volume replacement strategy in DKA. In this study, approximately 3500–4000 mL fluids were administered by the end of 6 h in both groups, and urine output exceeded 0.5 mL/kg/h, as recommended by the British Diabetes Society (Table 3) [3]. Moreover, urea and creatinine levels decreased (Table 1 and Table 3). Therefore, we agree with the recommended volume replacement strategy, provided that volume status evaluation methods such as delta–central venous pressure, arterio–venous carbon dioxide difference, the diameter of the vena cava, capillary filling time, and urine output are used together.

### 4.2. Elimination of Ketones

In DKA, the primary ketone produced is β-HB. Once β-HB is delivered from the liver to the plasma, it dissociates to β-HB^−^ and H^+^ [5]. β-HB^−^ load can be estimated by calculating the SIG level [4,7]. To extract β-HB^−^ from the urinary system, it should be bound to Na in the plasma. Thus, it makes sense to administer fluids with Na in DKA for the removal of β-HB^−^ from the urinary system. However, 0.9% NaCl, as suggested by the guidelines, contains equal concentrations of Na and Cl. Cl does not affect β-HB^−^ clearance and, in fact, induces hyperchloremic acidosis.

### 4.3. Correction of Electrolyte Imbalances

Hypokalaemia, the most common electrolyte imbalance in DKA, may occur due to osmotic diuresis and the uptake of potassium caused by keto anions. Insulin therapy also causes potassium to shift into cells. Replacement should maintain levels between 4 and 5 mEq/L, and close ECG monitoring is required in severe hypokalaemia. However, chloride is an underestimated electrolyte when correcting hyponatremia and hypokalaemia. Moreover, the guidelines recommend chloride-rich fluids [9]. This means that the guidelines’ fluid recommendation may lead to a new electrolyte imbalance—hyperchloremia. In this study, we observed hyperchloremia in patients administered chloride-rich fluids, although Na and K levels were similar in GI and GII (Table 3).

At this point, it seems that we need appropriate types of fluids that do not provoke new electrolyte disturbances.

### 4.4. What Is the Appropriate Type of Fluid?

Few studies in the literature examine the therapeutic effects of different crystalloid fluids on DKA and their impact on the development of side effects. The American Diabetes Association guideline recommends starting with 0.9% NaCl replacement for the first hour, then continuing with 0.45% NaCl according to sodium levels, but it does not clearly emphasize that chloride-rich fluids should be avoided [10].

In our data, the patients in both groups had similar metabolic characteristics at admission to the ICU; there were no differences in terms of chloride loads and they were in metabolic acidosis because of increased UA. However, at the end of 6 h in GI, Cl levels increased and BE_Cl_ decreased, markers of hyperchloremic acidosis.

It was noteworthy that the patients in GI were facing another metabolic acidosis iatrogenically in the most aggressive and dynamic phase of the treatment. In other words, in order to correct one type of metabolic acidosis, we created another type of metabolic acidosis, a situation that may be even more dangerous for such vulnerable patient groups. Interestingly, the American Diabetes Association guideline emphasizes that revisions can be made to the rate and volume of fluid resuscitation in patients with heart and kidney failure, and in elderly and adolescent patients, but there is no recommendation or revision protocol regarding the type of the fluid to be used [11].

Recent studies, particularly in paediatric DKA cases, have shown potential risks associated with fluid regimes while achieving these three objectives. In a study by Hay et al., it was observed that persistent acute kidney injury (AKI) and delayed recovery were associated with excessive intravenous fluid administration and hyperchloremia, highlighting the need for revision in DKA protocols [12]. Similarly, Parajuli et al. emphasize the effect of hyperchloremia on AKI, noting that it can worsen the clinical picture in patients who have suffered AKI due to hypovolemia [11]. In this case, a patient who is at risk of AKI due to hypovolemia upon arrival may face a greater probability of AKI with both excessive fluid resuscitation and hyperchloremia resulting from the replacements. On the other hand, restrictive use of chloride-containing fluids has been shown to reduce the development of AKI and the need for RRT treatment [13]. Although it has been stated as a negative result that lactated solutions may increase blood lactate values, recent studies suggest that balance solutions, especially lactated Ringer’s, may be associated with a faster recovery time in the treatment of DKA compared to 0.9% NaCl [14,15,16]. One such study showed that balanced solutions were faster in correcting DKA compared to 0.9% NaCl [17]. In a study comparing Ringer’s Lactate solution with normal saline administration in DKA, hyperchloremia and hypernatremia were significantly higher in the normal saline group. While no significant difference was found in the incidence of AKI, a much faster decrease in serum creatinine levels in the lactate Ringer group was noted, which was significant in terms of renal recovery [18].

In our study, although the intensive care unit (ICU) length of stay was short in both groups, it was longer in Group I. At 6 h, patients in the GI group were found to be more acidotic and hypocapnic, with lower bicarbonate levels. Similarly, we attribute the positive correlation between delta-Cl values and ICU length of stay to the delayed recovery of metabolic acidosis due to increased chloride load.

Therefore, based on our results, we consider it necessary to include chloride and BE_Cl_ in follow-up parameters to prevent undesirable metabolic consequences and to modify fluid choice as craftwork fluids. This approach differentiates our study from other previous DKA–fluid studies.

We are aware that our study has some limitations. First, our study is retrospective and has a small sample group. Second, no patient had advanced hemodynamic monitoring, so we were unable to examine the correlation between the depth of hypovolemic shock and acidosis. Thirdly, our study examines the effect of fluid therapy during the first 6 h on the development of acidosis as an outcome. Since patients in both groups improved and were discharged, we cannot make a positive comment regarding survival in relation to our fluid choice during the Golden Hours period. A study with more centres and patients would allow our data to draw stronger conclusions.

In the next phase of our study, we plan to investigate the correlation between BE_Cl_ values at 24 h and the end of ketonemia and organ failure. We hope that the inclusion of BE_Cl_ alongside standard blood gas monitoring may serve as a guide for individualised treatment protocols and the management of concomitant organ failure in DKA.

## 5. Conclusions

In conclusion, the administration of chloride-rich fluids in DKA may reduce UA acidosis by decreasing SIG but at the same time may cause iatrogenic hyperchloremic acidosis with a concomitant increase in serum chlorine and a decrease in BE_Cl_. Iatrogenic hyperchloremic metabolic acidosis may prevent the expected resolution of acidosis and hypocapnia, i.e., increased respiratory workload. Therefore, we believe that the type of fluid therapy in DKA guidelines needs to be reviewed. Individualised approaches should be brought forward by avoiding chloride-rich fluids and monitoring metabolic parameters such as chloride and BE_Cl_.

## Figures and Tables

**Table 1:** Comparison of patient characteristics and blood gas parameters at ICU admission.

	GI (Fluids with Na-Cl Difference = 0) (*n* = 22)	GII (Fluids with Na-Cl Difference > 32 mmol) (*n* = 13)	*p*
Patient characteristics			
Age (year)	52 ± 23	50 ± 18	0.567
Male (%)	10 (47.6)	4 (30.8)	0.477
BMI (kg/m^2^)	24.2 ± 3.2	26.6 ± 6.6	0.184
CCI	3 (1–7)	2 (2–6)	0.972
APACHE II	14 (11–24)	16 (12–25)	0.311
SOFA score	2 (1–6)	3 (1–9)	0.362
Blood gas and laboratory parameters at admission			
pH	7.10 ± 0.10	7.08 ± 0.10	0.562
PaCO_2_ (mmHg)	16.4 (8.1–31.7)	14.3 (6.5–22.6)	0.353
HCO_3_ (mmol/L)	7.2 ± 3.1	7.9 ± 3.5	0.577
SBE (mmol/L)	−24.7 (−28.9; −12.5)	−23.5 (−27.9; −7.8)	0.853
Na (mmol/L)	135 (120–146)	136 (124–138)	0.448
Cl (mmol/L)	106 ± 7	105 ± 6	0.876
BE_Cl_ (mmol/L)	−2 ± 6	−4 ± 3	0.337
K (mmol/L)	4.4 (2.8–5.6)	4.0 (1.9–7.6)	0.319
Lactate (mmol/L)	2.1 (0.7–9.1)	2.0 (1.3–8.6)	0.933
Hct (%)	40.9 ± 2.9	39.0 ± 3.0	0.067
Glucose (mg/dL)	426 (255–850)	537 (305–661)	0.801
SIG (mmol/L)	19.3 (11.1–33.4)	19.0 (7.0–24.7)	0.827
Urine ketones	3 (2–4)	2 (2–4)	0.649
Urea (mg/dL)	56 (13–132)	64 (49–115)	0.243
Creatinine (mg/dL)	1.5 (0.6–4.7)	1.9 (1.5–3.5)	0.086

APACHE, acute physiology and chronic health evaluation; BE_Cl_, base-excess chloride; BMI, body mass index; CCI, Charlton comorbidity index; ICU, intensive care unit; IMV, invasive mechanical ventilation; LOS, length of stay; SBE, standard base excess; SOFA, sequential organ failure assessment. Results are presented as mean ± sd, median (min–max), and percentage.

**Table 2:** Distribution of administered fluids in the first 6 h across groups.

	Na (mmol/L)	Cl (mmol/L)	Na-Cl Difference	GI (Fluids with Na-Cl Difference = 0) (*n* = 22)	GII (Fluids with Na-Cl Difference > 32 mmol) (*n* = 13)
%0.9 NaCl, *n* (%) (mL)	154	154	0	21 (95.2) 2302 ± 1188	-
%5 Dextrose %0.9 NaCl, *n* (%) (mL)	154	154	0	1 (4.6) 0 (0–600)	-
%0.45 NaCl, *n* (%) (mL)	77	77	0	1 (4.6) 0 (0–1500)	-
%5 Dextrose %0.45 NaCl, *n* (%) (mL)	77	77	0	4 (18.2) 0 (0–2000)	-
%5 Dextrose, *n* (%) (mL)	-	-	0	5 (22.7) 0 (0–1500)	-
Human Albumin 20%, *n* (%) (mL)	125	100	0	1 (4.6) 0 (0–60)	-
HES 6%, *n* (%) (mL)	154	154	0	1 (4.6) 0 (0–1000)	-
%0.45 NaCl (65 mEq NaHCO_3_/935 mL), *n* (%) (mL)	143	83	50	-	5 (38.5) 0 (0–4450)
Isolyte, *n* (%) (mL)	140	103	37	-	4 (30.8) 0 (0–3300)
Isolyte-S, *n* (%) (mL)	141	98	43	-	4 (30.8) 0 (0–3250)

Results are presented as mean ± sd, median (min–max), and percentage.

**Table 3:** Total fluid input, blood gas, and laboratory parameters at the 6th hour for both groups.

	GI (Fluids with Na-Cl Difference = 0) (*n* = 22)	GII (Fluids with Na-Cl Difference > 32 mmol) (*n* = 13)	*p*
***Total fluid input in the first 6 h***			
Administered fluid (mL/kg/h)	6.2 (4.4–15.5)	6.9 (5.3–10.9)	0.699
NaHCO_3_ (mmol)	60 (0–350)	100 (0–150)	0.389
KCl (mmol)	0 (0–75)	0 (0–30)	0.511
Insulin (U/kg/h)	0.08 (0.02–0.56)	0.08 (0.04–0.11)	0.933
Administered Na (mmol) (only from fluids)	343 (116–770)	420 (310–636)	0.091
Administered Cl (mmol) (only from fluids)	343 (116–770)	273 (216–374)	***0.016***
Na-Cl difference (mmol) (only for fluids)	0 (0–0)	144 (95–263)	***<0.001***
Urine output (mL kg^−1^ h^−1^)	3.6 (0.5–7.8)	2.7 (0.2–11.2)	0.448
***Blood gas parameters at the 6th hour***			
pH	7.28 (7.11–7.40)	7.38 (7.35–7.49)	***<0.001***
PaCO_2_ (mmHg)	23.0 ± 5.7	27.8 ± 3.5	***0.010***
HCO_3_ (mmol/L)	12.9 (4.3–21.9)	16.5 (16.1–23.1)	***<0.001***
SBE (mmol/L)	−14.6 (−23.2; 0.0)	−9.8 (−12.2; −2.8)	***0.002***
Na (mmol/L)	138 ± 6	139 ± 5	0.622
Cl, (mmol/L)	112 ± 7	104 ± 5	***<0.001***
BE_Cl_ (mmol/L)	−6 ± 5	3 ± 1	***<0.001***
K (mmol/L)	3.5 ± 0.6	3.2 ± 0.7	0.115
Lactate (mmol/L)	1.5 (0.8–6.4)	1.7 (0.9–7.4)	0.229
Hct (%)	35.9 ± 2.5	34.4 ± 2.1	0.091
Glucose (mg/dL)	256 (100–411)	268 (113–431)	0.972
SIG (mmol/L)	9.8 (−1.1; 22.8)	11.1 (−1.0; 15.7)	0.408
***Laboratory parameters at the 24th hour***			
Urine ketones (at the 24th hour)	2 (0–3)	1 (0–3)	0.302
Urea, (mg/dL) (at the 24th hour)	30 (16–100)	46 (34–92)	0.112
Creatinine (mg/dL), (at the 24th hour)	1.1 (0.4–3.9)	1.4 (0.9–2.6)	0.089
***Outcomes***			
LOS-ICU (days)	3 (2–4)	2 (1–3)	***0.023***

BE_Cl_, base excess chloride; SBE, standard base excess; SIG, strong ion gap. Results are presented as mean ± sd and median (min–max). Total fluid input in the first 6 h, Blood gas parameters at the 6th hour, Laboratory parameters at the 24th hour are Outcomes are presented as subheadings in the Table 3. Italicized *p* values indicate statistically significant results (*p* < 0.05).

**Table 4:** Multivariate linear regression model for LOS-ICU. R^2^: 0.66; Durbin Watson: 1.98.

	OR (95% CI)	*p*
Age	0.19 (−0.001; 0.039)	0.060
BMI	−0.37 (−0.097; 0.023)	0.214
APACHE II	−0.031 (−0.100; 0.039)	0.371
SOFA Score	0.068 (−0.087; 0.223)	0.373
Delta-pH (x100)	0.036 (−0.021; 0.092)	0.209
Delta-CO_2_	0.070 (−0.106; 0.156)	0.104
Delta-HCO_3_	−0.051 (−0.149; 0.047)	0.295
Delta-Na	−0.043 (−0.208; 0.123)	0.597
Delta-Cl	0.203 (0.021; 0.386)	*0.031*
delta-lactate	0.033 (0.020; 0.639)	*0.038*
Delta-SIG	0.069 (−0.097; 0.235)	0.396
Delta-glucose	0.001 (−0.002; 0.004)	0.602

LOS-ICU, length of ICU stay; SIG, strong ion gap. Italicized *p* values indicate statistically significant results (*p* < 0.05).

## Data Availability

The datasets for the current study are available from the corresponding author upon reasonable request.
